# Biocolonial pregnancies: Louise Erdrich’s *Future Home of the Living God* (2017)

**DOI:** 10.1136/medhum-2021-012250

**Published:** 2022-01-17

**Authors:** Anna Kemball

**Affiliations:** School of Literatures, Languages and Cultures, University of Edinburgh, Edinburgh, UK

**Keywords:** english literature, literature and medicine, medical humanities, women's health, pregnancy

## Abstract

This article argues that the health humanities must examine biocolonialism (and representations thereof) if it is to attend to Native American experiences of reproductive healthcare in the USA. Reproductive healthcare abuses are brought into dialogue with Native American resistance to Western biomedical sciences in *Future Home of the Living God* (2017) by Louise Erdrich (Ojibwe). Written over the course of two reinstatements of the Mexico City Policy, Erdrich’s novel invites a consideration of biocolonialism in relation to the exploitation and policing of female bodies.

After a discussion of bioprospecting and female bodies, I frame unethical practices of reproductive healthcare and sterilisation as biocolonial acts. The experience of the novel’s protagonist, Cedar Hawk Songmaker, will be situated alongside the broader ways in which Native Americans are subjected to surveillance. Second, this article proposes that speculative fiction allows for a temporal reframing of the colonial histories of Indigenous healthcare. As she narrates a world in which evolution ‘is running backward’, Cedar employs narrative reversals to resist the linear narrative of progress and ‘discovery’ associated with biomedical sciences.

The radically changing structures of a dystopian state, as well as the revelation of her biological inheritance, complicate the cultural and medical frameworks within which Cedar narrates her pregnancy. A challenge faced by the health humanities is how the discipline might theorise ongoing, interrelated forms of domination such as those which position female Indigenous bodies as ‘new colonies’. But, as I will argue, the mobilisation of Indigenous narrative forms and cultural frameworks offer productive directions for future work within the global health humanities.

## Introduction

As the health humanities extends the cultural and theoretical vantage points from which it critiques medical practice (Crawford *et al*. 2015; Hooker and Noonan 2011), and adopts a more politised form of critique (Whitehead 2014; Whitehead and Woods 2016), this article argues that an examination of biocolonialism is necessary to the exposition of global health inequities and injustices. Given that biocolonial theory relates more precisely to the exploitation of living organisms, natural resources and human bodies than decolonial or postcolonial modes of critique, a mobilisation of this criticism has much to contribute to the global health humanities. In return, a more sustained interrogation of health and health policy will develop the fundamental concepts found within antibiocolonial activism. Because the health humanities ‘enables and encourages fearless questioning of representations of caregivers and patients in all their varieties, [and] challenges abuses of power and authority’ (Jones, Wear, and Friedman 2014, 4), the field is well placed to shed light on biocolonial acts within healthcare settings of all kinds. Within this article, however, my focus shall be on Native American experiences of reproductive healthcare in the USA. This specialty particularly resonates with critiques of biocolonialism, given that anxieties surrounding reproduction and reproductive fitness have shaped, and continue to shape, national histories and social identities via neo-eugenic forms of population control (see Kluchin 2011; Reagan 2010; Sheldon 2016).1

Such anxieties have, in turn, become common tropes in speculative fiction, a genre that frequently depicts fertility crises in order to question the survival of future populations.2 Extending Gavin Miller and Anna McFarlane’s assertion that science fiction and other speculative genres offer ‘a valuable route to imagining socially just futures for medicine’ within medical humanities scholarship (Miller and McFarlane 2016, 217), this article explores the ways in which Indigenous speculative fiction exposes the injustices and discrimination experienced by minoritised populations.3 Daniel Heath Justice (Cherokee) argues that Indigenous speculative texts act as ‘humanizing interventions against the dehumanizing projections of those in power’ in settler colonial contexts (Justice 2018, 141). Although a central facet of biocolonial extractivism is the disaggregation of living organisms and natural resources through a disavowal of the vitality of Indigenous lifeforms, this literary genre reinscribes Indigenous peoples with sovereignty, relationality and agency in ways contrary to biocolonial logics. Indeed, parallels ought to be drawn between the ‘humanizing interventions’ made within literary critiques of biocolonialism, and a central pursuit in health humanities to ‘humanize’ medical practice (Jones, Wear, and Friedman 2014). Ultimately, by imagining health futurities that exaggerate current realities, Indigenous speculative narratives create spaces in which biocolonialism as a relatively recently theorised form of coloniality might be depicted and resisted.

This article shall examine how Louise Erdrich (Ojibwe) draws together reproductive healthcare abuses and Native American resistance to biocolonialism in *Future Home of the Living God*, a novel which charts the course of a Cedar Hawk Songmaker’s pregnancy amidst a global ‘biological confusion’ (Erdrich 2017, 5).4 As genetic mutations prevent animals from ‘breeding true’ (44), fears surround the reproductive capabilities of humans because of an apparently heightened risk of maternal or infant mortality during pregnancy and labour. This crisis prompts the very creation of Cedar’s epistolary narrative; for all that Cedar admits ‘lexical knowledge may be useless’ (3) on the first page of this diary, she documents ‘a record and an inquiry into the strangeness of things’ (62) for her unborn child. With human evolution feared to be ‘running backward’ (3), a newly established Church of the New Constitution declares martial law and a state of emergency in the USA. Every pregnant woman is required to submit to constant testing and biomonitoring, a policy of ‘gravid female detention’ (73). Once the crisis deepens, women are drafted as ‘Womb Volunteers’ by Mother, the Church’s public mouthpiece, and artificially inseminated with embryos that had been frozen at a time before genetic mutations might have taken hold. Under this totalitarian regime, where information is sparse and an organised resistance is only in its infancy, Cedar’s pregnancy unfolds. Written over the course of two reinstatements of the Mexico City Policy (the Global Gag rule)—responsible for drastically controlling the availability of reproductive healthcare across the world and perpetuating health inequities*—Future Home* invites a consideration of biocolonialism in relation to the exploitation and policing of Indigenous female bodies.

Erdrich’s foray into speculative fiction has been regarded as an experimental break from her earlier work, a ‘striking departure’ from other novels about Native American communities in North Dakota (Charles 2017). Yet framing the novel in this way, as a ‘departure’ from Erdrich’s other works of fiction, risks overlooking the author’s continued commitment to represent Native subjectivities and settler colonial practices in *Future Home*. A similar disjuncture between Erdrich and her representations of Indigeneity can be gleaned from initial reviews of the novel (Barclay 2020; Charles 2017), which have generally emphasised the thematic treatment of reproductive rights and control from feminist and ecocritical perspectives by drawing comparisons to Margaret Atwood’s *The Handmaid’s Tale* (Atwood 1985). As a figure who has been understood to embody sociopolitical concerns and revoked reproductive freedoms, Cedar has been framed ‘less as an individual than as the personification of *all* women whose bodies and choices are wrested from them’ (Brunjes 2018, 40; emphasis added). Relating Cedar’s subjectivity to a collective experience of stolen autonomy may offer a valuable entry point into this novel, but the tendency to universalise her experience, evident in the novel’s earlier reviews (Brunjes 2018; Charles 2017), comes at the expense of overlooking the specific experience and forms of subjugation enacted upon Native women. Moreover, ecocritical readings of Erdrich’s dystopia, while providing valuable analyses of the entanglements between evolution and extinction, risk doing so in ways that homogenise human culpability in these phenomena. Bridgitte Barclay’s framing of this novel as a ‘love letter to another species’ (Barclay 2020, 81) prioritises the anthropocentric destruction of non-human species, leaving scope to more fully explore the novel’s forms of domination enacted upon minoritised (human) populations. Following Audra Mitchell and Aadita Chaudhury’s recent exposition of the problematic claims to ‘universality’ in white apocalyptic discourses (Mitchell and Chaudhury 2020, 210), this article does not frame Cedar as representative of *all* women and does not compare her to white protagonists of corresponding feminist dystopias. Instead, I examine the processes of racialisation and the historic colonial practices that are elicited within this unspecified future time. Further, I demonstrate how interpreting *Future Home* through Indigenous criticism reveals where the treatment, surveillance and exploitation of Native women’s bodies undoubtedly become acts of biocolonialism. Second, this article proposes that speculative fiction allows for a temporal reframing of the colonial histories of Indigenous healthcare. As she narrates a world in which evolution ‘is running backward’ (3), Cedar employs narrative reversals to resist the overwhelmingly linear narrative of progress and ‘discovery’ associated with biomedical sciences. This reframing, which aligns with Indigenous conceptualisations of time, renders visible the relationship between biocolonialism and other forms of coloniality.

## Biocolonialism as a ‘new wave’ of colonialism

A reading of the colonial practices depicted in *Future Home* as specifically biocolonial is enabled through the ways in which fundamental critiques of extraction, ownership and commodification have been applied to the site of female bodies (Hawthorne 2007). Biocolonialism, as defined by Kooyooe Dukaddo (Northern Paiute) scholar Debra Harry, Executive Director of the Indigenous Peoples Council on Biocolonialism, is ’the control, manipulation and ownership of life itself, and the ancient knowledge systems held by Indigenous peoples’ (Harry 2014, 703). Extending colonial abuses of power within this ‘new wave of colonialism’, biopiracy and biocolonialism are understood as the exploitation and commodification of natural and genetic resources belonging to Indigenous peoples (Harry, Howard, and Shelton 2000, 7). Intellectual property rights, viewed by Harry as ‘part of the colonial arsenal of instruments of conquest’ (Harry 2014, 718), are used in order to legitimate private ownership, maintaining ‘a continuation of the oppressive power relations that have historically informed the interactions of western and indigenous cultures’ (Whitt 2009, 1).

To consider a dystopian novel about an international infertility crisis might, at first, seem at odds with the more established use of ‘biocolonialism’ as a form of oppression. But I would argue that the issues presented by Erdrich align with Laurelyn Whitt’s explanation of the ‘diverse forms of extractive biocolonialism’ in which ‘valued genetic resources and information are actively sought, ‘discovered’, and removed to the microworlds of biotechnoscience’ (Whitt 1998, 33). Once such resources become private intellectual property, they are ‘placed for sale in genetic marketplaces’ (Whitt 1998, 34). This process of discovery, removal and commodification maps onto the trajectory of Cedar’s pregnancy in *Future Home*: after strict policies of surveillance legitimate her capture, Cedar is sequestered in a prison-hospital where, if her baby survives birth, she is expected to surrender the child to a state which now highly values ‘original’ babies (245) unaffected by the evolutionary crisis.

Alongside research on biocolonialism within Native North America, Vandana Shiva’s research on biopiracy considers how ‘new colonies are being carved out’ on a global scale:

Capital now has to look for new colonies to invade and exploit for its further accumulation. These new colonies are, in my view, the interior spaces of the bodies of women, plants, and animals. (Shiva 2012, 5)

According to Shiva, reproductive healthcare and pregnancy should be understood as aspects of Indigenous life which are vulnerable to biocolonial exploitation. This projection of what, or who, will be targeted as ‘new colonies’ is combined with Whitt’s theorisations of extractive biocolonialism by Susan Hawthorne. Her examination of the extractive acts of biopiracy directly suggests that abuses in reproductive healthcare fall under the purview of biocolonial practices. Female bodies and Indigenous peoples, Hawthorne argues, have been colonised in ways analogous to the colonisation of land.

The mining of women’s body parts is routine and is especially widespread among those engaged in reproductive technologies and stem-cell and cloning research using the by-products of abortions. […] These latest assaults are an extension of the same philosophy. Women’s bodies are resources and a major site of colonization and profit making. (Hawthorne 2007, 318–19) 5

These various practices are, Hawthorne suggests, justified by the same colonial ideology; parallels can be drawn between the *terra nullius* principle which has historically ‘justified’ settler colonial occupation of ‘empty’ land and the fact that women’s bodies are ‘viewed as inert, passive and empty, that is, ripe for exploitation’ (Hawthorne 2007, 319). Hawthorne’s examination of bioprospecting and reproductive technologies, alongside Whitt’s extractive definition of biocolonialism, enables a reading of the colonial practices within *Future Home* as specifically biocolonial. Even if some instances of the practices described above are exaggerated within Erdrich’s speculative narrative, as shall be outlined later, the reproductive technologies employed are grounded within ongoing practices that resonate with Native American experiences of reproductive healthcare.

That biocoloniality and the exploitation of women’s bodies should be regarded as major sites of critique in *Future Home* is evidenced by the political climate which prompted Erdrich’s adoption of the speculative mode in her writing. While pregnant herself, Erdrich began writing *Future Home* in 2000, finding herself driven by President George W. Bush’s reinstatement of the Mexico City policy—referred to by critics of the policy as the Global Gag Rule (Atwood and Erdrich 2017). After years spent on separate literary projects, Erdrich returned to and completed *Future Home* in 2016 as President Donald Trump, like his Republican predecessor, reinstated the Global Gag Rule. When this policy is in effect, non-governmental organisations(NGOs) are not eligible to receive US federal funding if they use their own funding (or that of third parties) to provide access to abortions or abortion-related services, including counselling and education. The policy’s restrictions ‘effectively preclude such NGOs from providing safe abortion services even where it is legal in their own countries for indications not covered by the Gag’ (Crane and Dusenberry 2004, 128–29). Erdrich herself has described both the novel and policy in terms of reversal and regression: ‘we have a tendency to regress after we move forward. […] Maybe I’m writing the biological equivalent of our present political mess’ (Atwood and Erdrich 2017). As shall be discussed in further detail below, forms of reversal are foundational within Erdrich’s speculative reframing of healthcare abuses on Native women; society in a fictionalised USA collapses when evolution is understood to be ‘going backward’, reflecting the resurfacing of this contested foreign policy that revokes previously granted reproductive freedoms.

The Global Gag Rule (part of the ‘political mess’ to which Erdrich refers), was first enacted by President Ronald Regan in 1984. The policy, though rescinded by the Clinton and Obama administrations, has been reinstated by every Republican president. Trump’s expansion of the policy—renamed ‘Protecting Life in Global Health Assistance’—escalated the restrictions to all ‘global health assistance furnished by all [US] departments or agencies' (Executive Office of the President, Memorandum for the Secretary of State, Secretary of Health and Human Services and Administrator of the United States Agency for International Development (USAID) 2017, 8495), amounting to around $10 billion of US aid per year (Rodgers 2018, 2). This imposition of an antiabortion agenda on nations which have legalised abortion is regarded by Arianne Shahvisi as a ‘form of moral imperialism’ which violates the norms which generally govern donor-recipient relationships of foreign aid (Shahvisi 2018, 180; see also Crane and Dusenberry 2004). Further, Trump’s expanded policy meant that, if they provide comprehensive reproductive healthcare, foreign NGOs forfeited financial assistance for public health initiatives including treatment for the Zika virus, tuberculosis and HIV. Like his Democratic predecessors, President Joe Biden rescinded the Global Gag Rule in January 2021, broadly restoring the financial support that was available to foreign NGOs during the Obama administration. This decision has been welcomed by reproductive health organisations, but concerns remain regarding the long-term security of this funding and the lasting impact of Trump’s expanded restrictions (Lieberman 2021).

With such stark consequences for public health, the policy has been increasingly critiqued within anticolonial and global health discourse. No evidence has suggested that the number of abortions has reduced in countries where the Global Gag Rule is in effect. Rather, the limited available evidence collated by Jeffrey B. Bingenheimer and Patty Skuster suggests that the rule leads to an increase in abortions carried out, an increase in maternal mortality and negative child health outcomes (Bingenheimer and Skuster 2017, 280–82). That the policy was once again reinstated, despite evidence suggesting it fails to achieve its objective of reducing abortions (Rodgers 2018, 11), adds further credence to the notion that the Global Gag Rule is an imposition of political values which carries troubling ethical implications for human rights (Singh and Karim 2017, e387–e389).

Where the Global Gag Rule, ‘America’s deadly export’ (Filipovic 2017), undermines the autonomy of other nations to offer safe and legal abortions, health policy within the USA as it relates to Native American reproductive healthcare could similarly be understood within a neocolonial—specifically biocolonial—framework. The Hyde Amendment, passed in 1976 and annually renewed by Congress, currently prevents the use of federal funds for abortion services.6 Shaye Beverly Arnold’s research finds that the Indian Health Service (IHS) is usually ‘the sole provider of reproductive health services for Native populations’ (Arnold 2014, 1892). Funded at a federal level, the IHS is thus subject to the regulations stipulated by the Hyde Amendment: abortions can only be provided when pregnancies result from rape or incest, or when the mother’s life is threatened by the pregnancy. Ultimately, Arnold concludes that such restrictions ‘systematically infringe on the reproductive rights of Native American women’ (Arnold 2014, 1892; see also Theobald 2017, 231). Indeed, this systematic discrimination is an intensified example of how Indigenous motherhood is policed by the state (Gurr 2011).

Following the approach of Phillip Barrish, who argues that health humanities ought to address what he has termed ‘health policy fiction’, I have outlined the effects and implications of these policies on reproductive health because healthcare is ‘a complex set of financial models, public and private institutions, government policies, and actors whose roles range well beyond patient and care provider’ (Barrish 2019, 297). Epidemics are ‘catalyst[s] for social change’ (Reagan 2010, 5), and as illustrated within *Future Home,* though they often give rise to newly adapted health policies, they are also catalysts for new forms of stigma and new opportunities to demarcate or marginalise difference. Indeed, the ongoing COVID-19 pandemic exemplifies Reagan’s observations and highlights the urgent need for analytical practices employed by critics, including Barrish, that foreground the wider repercussions of health policy during times of global health crises.

## Reproductive healthcare, inheritance and surveillance in *Future Home of the Living God*

Erdrich’s speculative text presents the literary ‘afterlives’ of these health policies, and the colonial logics they uphold, by bringing together multifaceted concerns of inheritance and legacy in this time of a collective fertility crisis. To resist the new policies of surveillance and sequestration enforced by the Church of the New Constitution, Cedar goes into hiding, but not before establishing contact with her biological parents. As an ‘adopted child of Minneapolis liberals’ (3), Cedar Hawk Songmaker has only engaged with her Ojibwe identity in a limited way, regarding herself as a ‘theoretical Native’ (4).7, 8 Wanting to avoid ‘issues of abandonment and reconciliation’ (5), Cedar had not previously pursued any reconnection with her biological family. Now, her diary reveals that any notion of cultural reconciliation—recovering a lost community or cultural heritage—is replaced by the seemingly more pressing matter of biogenetic inheritance when, prompted by the health crisis, Cedar contacts her birth mother.

I wouldn’t have had the slightest thing to do with them if it wasn’t for my baby. […] You deserve more. You deserve two sets of grandparents. Not to mention genetic info, which may affect who you are even beyond whatever is now occurring. There may be hereditary illnesses. (6)

Concerned exclusively with the genetic information, rather than Native ‘culture’, that the baby might inherit from their ‘bio-based grandparents’ (4), Cedar’s actions and priorities mirror those of the state, which foregrounds biogenetic security and fetal health above all else. The biological and reproductive continuance of the general population motivates new totalitarian measures at the expense of other aspects of cultural and social welfare. But the apprehension surrounding hereditary illnesses within Cedar’s biological family is conflated with a wider biogenetic threat faced by her unborn child. While this concern might seem futile within the wider context of a global pandemic, the determination to establish the health of her child at the level of individual inheritance parallels the purpose and function of her construction of a written record of the pandemic for an individual reader.

Throughout the diary, Cedar figuratively constructs her child as a reader. This is primarily achieved by narrativising stages of fetal development with second person pronouns: ‘your brain is hooked up to stereo – your ears. […] You can hear me as I read aloud the first words of my letter to you’ (63). Although Cedar is aware that constructing this narrative is of limited use in such turbulent times, addressing her prenatal reader becomes, in this state of uncertainty, an important means of willing her baby’s survival and the hoped-for end of the evolutionary pandemic. Unsure of the baby’s health during this crisis, Cedar imaginatively creates a healthy reader who, years later, is able to read their mother’s diary. As observed, however, within Rebekah Sheldon’s comprehensive analysis of the child figure in ‘sterility apocalypses’, a ‘conjunction of the child with the future is never clean, […] because the child is not a guarantee of replication’ (Sheldon 2016, 35). This ‘big assumption, maybe wishful thinking’ (166) that Cedar’s child will survive birth and retrieve this written record plays a vital role in what Silvia Martínez-Falquina has termed as Erdrich’s ‘aesthetics of uncertainty’, a style which renders visible the novel’s various forms of vulnerability and which requires the reader’s ‘active participation in the construction of meaning’ (Martínez-Falquina 2019, 165; 167). Indeed, this diary quickly becomes a legacy gifted to the child in conjunction with their (presumably ‘healthy’) genetic inheritance. Although Martínez-Falquina frames the participation of Cedar’s reader in terms of vulnerability, narrating her child’s future readership into the text itself is one of the few ways Cedar can escape the escalating powers of the new government, a characteristic tactic of ‘fully empowered narrators’ within dystopias who, David W. Sisk notes, ‘control the shapes of their stories even though they control nothing else’ (Sisk 1997, 176). Implied within this act is Cedar’s radical hope that the evolutionary crisis and totalitarian regime might have ended (in that she assumes this will be something her child has to learn *about* rather than experience directly).

The refuge that Cedar, as narrator of her child’s future health, accesses through writing outlives her physical safety once her pregnancy is made known to the Church of the New Constitution. Phil, the father of her child, is arrested and tortured by ministers during a ‘truth seminar’ (191) before revealing Cedar’s location. Captured by the Unborn Protection Society, an organisation tasked with tracing and detaining pregnant women (and which have repurposed the United Parcel Service’s vehicles), Cedar is taken to Fairview Riverside Hospital to ‘give birth under controlled circumstances’ (72). It is in this setting that Cedar’s body is most fully treated as a ‘new colony’ (Shiva 2012, 5); patient consent is never sought by healthcare professionals who withhold fundamental information about the health of herself and her child. Sedatives are administered under the falsehood that the women must take ‘vitamins’ so that the imprisoned, medicated patients are made compliant. Within a strict regimen of daily biomonitoring, blood, urine, hair and nail clippings are extracted and catalogued by nurses under the assumption that new data about the ‘biological confusion’ (5) might be obtained. In their discussion about biomonitoring in the twenty-first century, specifically of breast milk, Monica J. Casper and Lisa Jean Moore explain how such extractive surveillance allows liquids, tissues and body parts to ‘stand in for and […] represent whole persons – indeed, entire communities’ (Casper and Moore 2009, 110). They continue:

It is clear that our fluids, as taken from us and used for evaluative purposes, are unpredictable and leaky. They become useful to others – organisations, the state, and forms of governmentality – and then are made to tell certain kinds of biopolitical truths about our bodies. […] Our bodies are no longer seen to matter as individual lives, but, rather, our fluids and tissues taken together represent a kind of environmental aggregate that speaks volumes about the future of our species on the planet. (Casper and Moore 2009, 118)

Tellingly, Casper and Moore are aware that such practices hardly seem believable: ‘These parts, then, as if in some science fiction dystopia, achieve a monstrous power over us in the form of numeric codes’ (Casper and Moore 2009, 118). It is hardly surprising, then, to see such practices reflected back within Erdrich’s dystopian representations of biopower over subjugated bodies. Although such disaggregation—of blood, breast milk and other fluids—is not termed as biocolonial by Casper and Moore, elsewhere extractivism has been identified, in both practical and ideological terms, as fundamental to settler colonialism (Elliott 2019; Simpson 2017). Indeed, these practices reflect ‘the settler presumption that all things Indigenous are ripe for the taking’ (Justice 2018, 138), be it Cedar’s blood, her body, her freedom or, ultimately, her child. Justice’s analysis of this settler logic culminates in a discussion of medical experiments carried out within Canada’s residential school system (Justice 2018, 135–139). Equally, by combining such forms of biomonitoring and the forced extraction of biological material with the anticipated postpartum removal of a child, Erdrich brings together the interrelated forms of coloniality that have, for centuries, enacted violence on Native bodies and dismantled Indigenous social structures.

Moreover, the underlying threat that Cedar may not keep her child, having heard the rumours that hospitals keep surviving babies, reflects the structures of institutional and private ownership which enable biocolonial acts to occur. Indigenous knowledge and biological material are converted into private property by the dominant culture to maintain a relationship of colonial control over Indigenous peoples (Whitt 2009, 23). Undoubtedly, Cedar’s subject position as a transcultural adoptee—a woman who doesn’t ‘stand out as Native unless people already know’ (51) and who later learns that her father *is* indeed white—complicates a reading of the novel that would seek to clearly distinguish between the dominant and oppressed group(s) within whose relationship of dominance such biocolonialism might occur.9 Nevertheless, Cedar’s position, together with Erdrich’s weaving of biological, cultural, evolutionary and political inheritances in fact parallel the ‘complex webs of power’ at play within biocolonialism, emulating ‘the intersecting strands of which buttress and strengthen one another’ in order to assert sovereignty over Indigenous peoples (Whitt 2009, 20).

Despite Cedar’s position as an adoptee who can pass as white, racialisation does occur within clinical settings during her pregnancy, entangling the dual structures of race and reproductive capability which now demarcate the population under the Church of the New Constitution. Processes of racialisation and racial discrimination are less overt than the imprisonment of pregnant women or conscription of ‘Womb Volunteers’, yet fragments of information, documented within Cedar’s diary, suggest that white superiority is reinforced during this crisis. Cedar receives such information from a doctor-turned-informant during an appointment in the early stages of her pregnancy. At first, this ultrasound scan is indicative of the coercive or non-consensual clinical encounters faced by every pregnant woman under the regime. Technicians ignore Cedar’s questions as they measure the fetal skull, vertebrae and limbs. The ostensible viability of her pregnancy, ‘evidenced’ by the doctor’s interpretation of these measurements, means Cedar will not be allowed to leave the clinic of her own volition; medical institutions must now sequester and monitor every woman displaying signs of a ‘normal’ pregnancy. When left alone with Cedar, however, the unnamed doctor instructs her to restrain him and escape. Before the doctor is voluntarily gagged, he asks if Cedar has ‘any special ethnicity’ (51).

‘Yeah, I’m Ojibwe,’ I tell him.

He asks me about the father, is he white?

‘As milk,’ I said.

‘Then get the hell out of here. […] When you get out, don’t tell anybody that you’re pregnant.’ (51)

Even as she is in the very process of escaping, the doctor emphatically instructs Cedar to ‘get the hell out of here’ once he learns that the child’s father is white. This would suggest that race is a contributing factor in how the regime assigns value to the pregnancies it monitors and, more broadly, confirms Brianna Theobald’s argument that, for Indigenous women, ‘reproductive politics cannot be disentangled from the structures of white supremacy and capitalism’ (Theobald 2017, 222). Indeed, the increased ‘value’ in *Future Home* of a child of Indigenous and white parentage aligns with longer histories of forced child removal and cultural assimilation in settler colonial contexts which privilege whiteness. Given the restricted narrative perspective and limited information Cedar can access, readers cannot know the full extent to which whiteness is privileged by the Church of the New Constitution, or how this clinical encounter would unfold for prospective parents of other races or ethnicities. But the doctor’s implication that Cedar is in urgent danger, as a white-coded pregnant woman carrying the child of a white father, affirms the white supremacist logics on which forms of biocolonial extraction are predicated.

With the ensuing panic surrounding the future of the population—specifically, the white population—a ‘desperate market’, to use Rachel Stein’s phrase, might materialise in relation to the increasing value of pregnant bodies and others who could carry viable fetuses (Stein 2010, 104). In her work on cinematic portrayals of biocolonialism, Stein notes that the rush of such desperate markets to find effective medical treatments can lead to ethical breaches. Stein catalogues various breaches which are exaggerated by ‘the disparity in knowledge of medical practices between testers and test subjects’, including coercive testing practices, lack of informed consent and the heightened risk present during hastened drug trials (Stein 2010, 104). Departing from the cinematic depictions analysed by Stein, the geopolitical divisions between consumers, healthcare professionals and test subjects are not drawn across national borders in Erdrich’s US-based narrative. Yet a racialised hierarchy similarly alludes to the commodification of minoritised patients. Cedar’s diary gives voice to her Ojibwe family’s fear that, within a generation, the ‘wealthiest will get ahold of the technology to reproduce’ (95), forging a new modality of subjugation based on fertility.

Towards the end of the novel, the value placed on Cedar’s unborn child appears to increase. Once she is reunited with Phil, Cedar learns that she is now classified as ‘carrying one of the originals’ (245). In other words, given the time of conception, her baby might not be affected by the evolutionary crisis. When combined with their highly prized ethnicity (as divulged by the informant doctor), the ‘originary’ status of the baby apparently carries significant monetary value.

‘The thing is,’ he says, very softly, ‘you have a treasure, Cedar, if our baby is normal. We could be in charge of things. Rich. Super rich! We’d be safe. If we somehow worked out genetically, I mean, to have a normal child the sky’s the limit for us.’

‘We could seize power and found a dynasty,’ I say, meaning it sarcastically.

‘That’s right,’ says Phil softly, reaching for me. I bat away his hand and call Eddy. (246-247)

Phil’s speculation suggests that a political elite has emerged based on reproductive capability, infant survival rates and a biomedicalisation of normalcy. Where Cedar’s sarcasm is explicit, Phil’s motives are less easy to ascertain as he reaches for the mother of his child, the carrier of a potential source of wealth and prosperity required to survive within this regime. Cedar’s narration of the conversation within her diary hints towards a desire to capitalise on the ‘normality’ of their child and the privilege it would afford him. The extent to which this can be framed as biocolonial, in systemic terms, is obfuscated by Phil’s relationship to his unborn child and the hypothetical nature of the conversation. Nevertheless, Cedar instinctively remains ‘alert to something bad beneath his words’ (246) as Phil imaginatively places himself within the elite group. In this way, the future that Phil imagines upholds the commodification of profitable pregnant bodies, alongside the ostensible value placed by the state on whiteness and normalcy.

## Indigenous speculative fiction: ‘We wouldn’t see the narrative we think we know.’ (55)

I have relayed how the fertility crisis at the centre of *Future Home* allows for biocolonial practices to occur within reproductive and antenatal healthcare. In this section, I explore how Erdrich’s deployment of the speculative genre allows for a temporal reframing of colonial histories as they relate to reproductive healthcare. Sheldon has similarly forged interpretative associations between the temporally disjointed figure of the child in speculative fiction, and the genre’s potential for ‘textual reproductions’: encompassing depictions of birth and the narrative mechanisms by which futurities are made possible through a ‘novel’s structural rejection of reproduction’ (Sheldon 2016, 57–58). Specifically, I will discuss Erdrich’s mobilisation of Indigenous temporalities within the structure of her text, locating this within an increasingly speculative turn within Indigenous literature as ‘the uses and abuses of Indigenous bodies as a way of breaking generational bonds’ are narrativised in settler colonial futures (Justice 2018, 137). As he discusses Indigenous narratives of the apocalypse, Justice suggests that Indigenous writers ‘think about what endures *beyond* it, and they imagine the living, loving, and connecting that takes place in the ruins of settler colonial excess’ (Justice 2018, 167). But what of a narrative that appears to take place *in the midst* of an apocalypse? Given the diary’s form and Cedar’s limited narrative perspective, it seems impossible for Cedar or the reader to truly know what lies *beyond* the evolutionary crisis. Yet her present-tense narration of an epidemic, created in the hope that it might archive an account to be later read by her descendants, reveals a comparable concern for Indigenous futurity. Rather than projecting ahead to a postapocalyptic imaginary, as envisaged by Justice and various proponents of Indigenous futurism, Erdrich instead proposes a series of reversals which deliberately mimic the very evolutionary reversals that have precipitated the crisis.10

Contrary to the control exerted over her body and pregnancy, moments where Cedar faces intense danger are often entries within the diary where she displays the most agency as a narrator. Erdrich pairs the evolutionary reversals with the withdrawal of reproductive freedoms, and with reversals in the story-telling process itself. Condensed across a few diary excerpts, reversals are enacted across these three facets in *Future Home*. On 18 November, Cedar demands answers from her adoptive mother, Sera, about the nature of her adoption. In a blunt exchange, Sera announces that Glen is in fact Cedar’s biological father, having met Mary Potts while legally representing the tribe. So that Sera would never be regarded as ‘the possibly lesser ‘adoptive’ parent’ (234), the Songmakers agreed to raise Cedar under the guise of a full adoption. Cedar’s body physically reacts to the revelation, but not before her narrative voice immediately states, ‘What a weird reversal. Impossible to take that in’ (232). Despite Cedar’s previous embrace of her adoptive identity and multifaceted heritage, she frames these ‘sudden reversals in (her) own narrative’ (238) as a loss: ‘If that’s true, if you kept that from me, I could have had my real father all my life.’ (233).11

Following this dismantling of her adoptive identity, which places *Future Home* within a body of literature which complicates the dominant paradigms through which we culturally understand adoption practices (see Novy 2005, 7), a reversal occurs within the narration of Cedar’s diary. In her own words, an ‘unnecessary deception’ (234) had been put in place to conceal her biological relationship to Glen; now, the reader is similarly deceived as they question the truthfulness of the text. The reader is told that on 20 November an armed group, led by Mother, forced entry into the Potts’ home. This suggests an escalation of the state’s ‘gravid female detention’ policies (73); Mother, the regime’s mouthpiece who had previously practiced virtual surveillance by overriding Cedar’s computer, now physically searches for Cedar in person.12 Hidden under a pile of clothes, Cedar evades capture but is left confused: ‘I immediately decide that I did not see what I did see’ (240). In this disorienting passage, Phil is revealed as ‘one of Mother’s helpers’ (242) who, after the unsuccessful raid, proceeds to capture and transport the mother of his child. A belief in the veracity of Cedar’s narration slips from the reader’s grasp as contradictory statements emerge:

He is the father of my baby and I start to cry, silently, my face in my hands, the tears popping out and rolling down my fingers.

No I don’t. I sit in the heated car seat, dry-eyed, outraged. […] Phil. Another angel of deception. (242)

After the 20 November entry, the subsequent Thanksgiving entry (one of the few chapters without a precise date) calls into question what the reader has just been told. Moments of confusion are expanded, exposing the full extent to which the reader is dependent on and perhaps manipulated by Cedar’s narrative.

There never was a raid. Mother did not come back. Perhaps I hallucinated her. And maybe, let’s hope, in the throes of eighth month’s hormones I hallucinated Phil. But no, Phil was out the door once I called Eddy. (247)

A justification for this reversal of narrative events is never given, but two related reasons arise. First, this demonstrates Cedar’s agency over the narrative as a ‘fully empowered’ narrator of a dystopian text who has control solely over the shape of their story (Sisk 1997, 176). Exaggerations of Mother as a threatening presence exemplify Cedar’s exerted control over her narrative at a time when other forms of agency have been relinquished by the state. Second, the narrative strategy undermines the legitimacy and longevity of this totalitarian regime: Cedar can falsify the danger before ‘removing’ their policies of surveillance and capture from her diary. Notably, this narrative reversal takes place within the Thanksgiving entry; Cedar reveals her own processes of fictionalisation on a day which celebrates settlement by mythologising and pacifying oppressive relations between settler colonialists and Native Americans.

Kyle P. Whyte (Potawatomi) has researched Indigenous dystopian fiction in relation to climate crises. He shares knowledge passed to him from Anishinaabe elders of ‘intergenerational time’ and ‘spiralling temporalities’ (Whyte 2018, 228). Such temporalities are an integral part of many Indigenous cultures, but it’s also key to stress the specificity of this Anishinaabe perspective, given Cedar’s subject position and Erdrich’s own tribal affiliation. Whyte identifies ‘narratives of cyclicality, *reversal*, dream-like scenarios, simultaneity, [and] counter-factuality’ as examples of how spiralling time might be experienced (Whyte 2018, 229; emphasis added). Whyte argues that a mobilisation of these narrative temporalities foregrounds Indigenous science and knowledge because experiencing spiralling time involves participating ‘within living narratives involving our ancestors and descendants’ (Whyte 2018, 229). *Future Home* is not a novel that deploys Indigenous sciences in great detail, but the textual reversals in Cedar’s diary (written for her relatives) offer a means of resisting colonial oppression given, as Whyte observes, their relation to Indigenous knowledges.

The mutability of unfixed threads of narrative across the diary’s Thanksgiving entries resonates with the story of Cedar’s adoption, as well as the literal function of the unravelled and rewoven thread that had facilitated Cedar’s escape from the prison-hospital. Writing of transcultural adoption memoirs, John McLeod finds examples of adoption stories which ‘chang[e] their ending so that surprises lurk up ahead’, mirroring the continual flux of belonging and interstitial space of racial identification between self, family and the world (McLeod 2015, 229). Notably, Mcleod returns to textile metaphors of knots and woven threads to convey kinship beyond biocentric identity models. This aligns, too, with the themes of ‘rescue and captivity’ analysed in narratives of Native American adoption by Cynthia Callahan. These themes carry multiple ‘attendant meanings that are informed by cultural history’ and undoubtedly complicate the different modes of escape at play once Cedar’s adoptive identity unravels (Callahan 2011, 110). The possibility of escape offered by Cedar’s reversed narration textually reproduces the mode of unravelling and weaving employed by Spider Nun, Cedar’s roommate within the hospital.13 For several weeks, Spider Nun and Cedar meticulously pluck and unravel yarn from a supply of hospital blankets, before secretly reconstructing the yarn into rope with which they might scale down the building. Cedar likens Spider Nun’s way of weaving the hospital blankets to the ‘[o]ld-time’ Ojibwe methods (141) of finger weaving used by her grandmother, Mary Virginia, to create a baby carrier for Cedar’s child. The subversive processes of unravelling and rearranging Cedar’s narrative through such reversals, then, draw on Indigenous spiral temporalities and Ojibwe artistic practices.14 Together, these thematic, textile and narratives modes of unmaking counter a biocolonial regime of forced replication, thus endowing Erdrich’s speculative text (an otherwise linear diary which progresses towards her due date) with cyclical temporalities that offer, to a degree, the possibility of an escape.

## Sterilisation abuse and the IHS

Returning to how the novel references the colonial exploitation of Native American women and unethical abuses of reproductive healthcare reveals another sustained mobilisation of this reversal motif. Erdrich notably modifies how the theme of reversal has generally been deployed by other Indigenous writers who expose white anxieties of loss and assert plural sovereignties via ‘the reversal of trajectories of oppression’ (Mitchell and Chaudhury 2020, 325). As observed above, Cedar’s narrative can unwind and rearrange certain forms of oppression, but the generic limitations of this diary mean that a total undoing of these trajectories cannot occur. Instead, Erdrich employs reversals in an oppositional sense, critiquing sterilisation abuses endured by Native American women in the twentieth century by depicting oppositional forms of subjugation in this dystopian society.

Rebecca M. Kluchin’s enquiry into neo-eugenic policies during the 1960s and 1970s finds that, in the USA, women of colour ‘struggled to resist coercive sterilization’ (Kluchin 2011, 8). Guided by the assumption that restricting birth rates on Native reservations would reduce dependence on government assistance, IHS physicians and other services, contracted by the IHS, sterilised between 25% and 42% of Native American women of childbearing age during the 1970s (Kluchin 2011, 7; Lawrence 2000). Several factors call into question the voluntariness of these procedures and instead point to widespread practices of sterilisation abuse. Sally J. Torpy maintains that a lack of interpreters required to explain information in a patient’s first language constituted a frequent violation of Native American women’s rights (Torpy 2000, 13). Further coercive practices were conducted which denied the reproductive freedom of these women: the irreversibility of sterilisation was not always explained, other forms of contraception were not always offered, consent was garnered from already medicated patients, and women were threatened with the removal of existing children if they did not comply. The scale of sterilisation abuse amounted to ‘the loss of a generation of children’ (Vicenti Carpio 2004, 50), a population reduction which is believed by some tribal advocates to have been enacted so that the federal government might encroach further on Native American land as populations decreased (Kluchin 2011). According to Jane Lawrence, ‘[t]ribal communities lost much of their ability to reproduce, the respect of other tribal entities, and political power in the tribal councils’ (Lawrence 2000, 411). Furthermore, the reduced birth rate, which for Native American women fell at a rate ‘seven times greater than that of white women’ during the 1970s (Ralstin-Lewis 2005, 72), affected the sovereignty and economic base of affected tribes. Though Native American women were not the sole victims of coerced sterilisations during this period, this particular association between sterility, land encroachment and a weakened sense of political sovereignty affirm the biocolonial logics on which this strategy was based.

The 1976 Indian Healthcare Improvement Act saw greater Native participation within and control over IHS facilities. This, together with regulations introduced in 1979 by the Department of Health, Education and Welfare designed to prevent coercive sterilisations, did bring about a reduction in sterilisation abuse (Lawrence 2000). More difficult to eradicate, however, are the ‘attitudes that perpetuate the subjugation of women’s bodies’ (Vicenti Carpio 2004, 51). Certainly, the persistence of such attitudes is recognised by critics of the Global Gag Rule and the Hyde Amendment who identify a continual restricted access to full reproductive healthcare on the basis of ethnicity or nationality (Arnold 2014; Gurr 2011; Shahvisi 2018).

Erdrich’s most effective thematic ‘reversal’ of these widespread sterilisations appears in *Future Home’*s concluding sections. Despite previous evasions of state authorities, a heavily pregnant Cedar is kidnapped and confined in a prison that has been converted, even if in name only, into a ‘birthing centre’:

A sign above the entrance says Stillwater Birthing Center, but it is only a painted piece of canvas that covers Minnesota Correctional Facility Stillwater. (249)

It is within this institution that Erdrich portrays the full scale and violence of institutionalised biocolonialism as it might relate to pregnant women of colour. Women are cyclically inseminated and forced into pregnancy, with one inmate currently ‘carrying baby number three’ (252). When detainees use hunger strikes as a non-violent mode of protest, force-feeding methods are deployed by guards. Among women ‘of every colouring and age’, Cedar is racialised as a Native American woman in Stillwater Birthing Centre because she was captured in possession of her newly acquired form of identification. On the one hand, Cedar—now ‘Mary Potts with a tribal ID’ (249)—can evade detection for the crimes she committed to escape from the hospital (that is, within the regime’s model of criminality). Yet, that she is institutionally recognised as a minority subject now reinforces a biocolonial reading of her treatment within the birthing centre. Mandatory viewing during ‘Watching Hour’ (254) of religious propaganda purports that the women are only sentenced for the duration of a single pregnancy. Cedar firmly believes, however, that she will be imprisoned for longer: ‘Nobody gets out of here in 9 months. I’m positive. Nobody gets out of here at all’ (255). Once her baby is born, Cedar is rendered unconscious and the child is removed from her arms. From there, the health and the whereabouts of Cedar’s child is never known. As the shortest entries of her diary, two lines describe the end of December and the entire month of January: ‘Extremely weak. But still here’; ‘They say my heart is damaged’ (265). When her diary resumes in more detail, readers are told that Cedar awaits her ‘next pregnancy’ (266), the implication being that—because she has survived labour—she is a viable candidate for further inseminations. Given this continued confinement and the generic limitations of this epistolary narrative, Cedar cannot provide an account of her freedom, or the resolution of this ‘biological confusion’ (5). Although Cedar is able to imaginatively reconstruct and archive memories from a time before the crisis took hold in this diary, Erdrich’s narrative ends in a way that maintains biocolonial abuses within reproductive healthcare as a continuing phenomenon.

## Conclusion: dystopian healthscapes and adaptation

A challenge faced by the health humanities is how the discipline might theorise and critique ongoing, interrelated forms of domination such as those which position female Indigenous bodies as ‘new colonies’ (Shiva 2012). If critics working within the health humanities were to become more attuned to the practices of biocolonialism, we would become increasingly aware of how it is built on historical and existing colonial practices. As observed in *Future Home*, ‘Indians have been adapting since before 1492’ (28). Working within a speculative genre which allows for a temporal reframing of colonial oppression, Erdrich presents a dystopian society that seems to be a fictionalised future but is in fact a biocolonial rendering of the historical and ongoing injustices experienced by Native American women. Like Erdrich’s characters, our methods of reading ‘health policy fiction’ (Barrish 2019), of theorising representations of biocolonialism and of relating to Indigenous scholarship within the health humanities must likewise demonstrate this capacity to adapt. This article has offered a reading of biocolonialism as it relates to reproductive healthcare in one work of Native American fiction. But I want to conclude by highlighting the possibilities presented by *Future Home’s* interwoven concerns; the mobilisation of Indigenous narrative forms within *Future Home* offers productive directions for future work within the global health humanities and invites future analysis of Indigenous literatures.

As if this were not already an urgent task, the nexus between health, bodily autonomy, settler colonial governance and Indigenous sovereignty as exposed by the COVID-19 pandemic surely points to this necessary undertaking.15 Engaging with cultural responses to policies which impact on health jurisdiction and health outcomes of minoritised communities must be prioritised as a global health humanities undertake work on biocolonialism and the COVID-19 pandemic alike. Imagining how socially just futures of health will arise is essential to those for whom such dystopian healthscapes resonate with historic and continuing forms of oppression.16 Further, the Native strategies of resistance and narrative reclamation of agency exercised by Cedar underline the ways in which biocolonialism might be challenged. Sisk has stated that ‘a dystopian work fails if it does not move its reader to compare his or her ‘real world’ to the fictional society and consider how the latter could arise from the former’ (Sisk 1997, 9). Although, as I have argued above, the world presented in *Future Home* is not as fictionalised as it would first appear, it would follow Sisk’s line of thought to consider how Erdrich’s novel sheds light on practices in reproductive healthcare and restrictive policies which continue to constrain the reproductive freedoms of Native American women. While alternatives to regimes such as the Church of the New Constitution are not imagined in their entirety within *Future Home*, Cedar’s narrative certainly presents strategies which dismantle and undermine the oppressive and inequitable medical realities currently experienced by Native American women.

## Data Availability

No data are available. No data sets were generated and/or analysed for this study.

## Notes

1. I thank one of the anonymous reviewers of this article for recommending Sheldon’s research.

2. My use of the term ‘speculative fiction’ follows Sami Schalk’s usage, referring to creative writing where ‘the rules of reality do not fully apply, including magical realism, utopian and dystopian literature, fantasy, science fiction, voodoo, ghost stories, and hybrid genres’ (Schalk 2018, 17).

3. Following the practice of a growing number of scholars within Indigenous studies, I capitalise ‘Indigenous’ and ascribe a proper noun in order to affirm the political status of Indigenous peoples (see Justice 2018). I provide tribal affiliations when referring to individual authors and scholars where this information is available to me. I will use the term Ojibwe throughout, in keeping with the self-identifications of both Erdrich (an enrolled member of the Turtle Mountain Band of Chippewa) and Cedar as a narrator.

4. Subsequent quotations from Erdrich’s novel are indicated with in-text page references.

5. Hawthorne’s research also includes people with disabilities, an intersection that lies beyond the scope of this article. For more on the commodification of women, specifically how reproductive technologies have made women’s bodies ‘productive’ in ways that render them vulnerable to global marketisation, see Hawthorne (2002).

6. At the time of writing, the Hyde Amendment is still in effect. Scrutiny of this policy increased during the 2020 presidential election as President Joe Biden denounced the amendment (Glueck 2019). Biden proposed a 2022 spending bill without the Hyde Amendment that passed the House of Representatives in July 2021.

7. From the outset of her diary, Cedar is suspiciously aware that the 1978 Indian Child Welfare Act should have applied to her; tribal courts should have held jurisdiction over her adoption. The evasiveness of Sera and Glen Songmaker, Cedar’s adoptive parents, on this matter casts suspicion over the true nature of her relationship to them. For a comprehensive discussion of the ICWA, see Hollinger (1989).

8. Cedar’s full name is given by her white adoptive parents, perhaps to convey the stereotypical ‘hotline to nature’ (5) associated with Native subjectivity. By contrast, her birth name, ‘Mary Potts’, connects Cedar to several Ojibwe relatives who share this name.

9. Another facet of Cedar’s character that resists simplified interpretation is her devotion to Catholicism. Although beyond the scope of this article, Barclay (2020, 75–76) and Martínez-Falquina (2019, 172–174) discuss Cedar’s Catholicism in further detail.

10. In 2012, Grace L. Dillon (2012) (Anishinaabe) coined the name ‘Indigenous futurism’, referring to the theoretical approaches and cultural movements which imagine times and spaces that are both outside and beyond settler supremacy. Relatedly, Indigenous futurism challenges settler colonial understandings of what might be understood of as advanced civilizations and advanced technology. While *Future Home* engages with Indigenous futurities, central characteristics of Indigenous Futurism are not present; for example, Erdrich does not imagine ‘a continuation of a spiritual and cultural path that remains unbroken by genocide and war’ (Dillon 2016, 2).

11. Deans (2004) has comprehensively analysed the significance of adoption within Erdrich’s earlier novels, situating the trope in relation to Erdrich’s own role as an adoptive mother.

12. Livia Arndal Woods’ reading of the ‘inscrutable’ reality of Mother questions whether this surveillance figure might be a hallucination (Woods 2017). While indeed, Mother’s presence resists fixed interpretations, a biocolonial reading illustrates how Mother connects to longer histories, and ongoing practices, of surveillance and policing as experienced by Native American women. Whether hallucinatory or not, a reading of Mother, presented as she is in a novel which pointedly resists realism, should be understood within the oppressive continuum of colonial and paternalistic practices.

13. Spider Nun is in fact named by Cedar for her constant weaving and her complete muteness during the hospital imprisonment (after their escape is her name given as Tia Jackson). Deans notes the significance ascribed to the image of a spider’s webs when Erdrich writes about adoptive parenthood, given that the web is ‘both constructed and spun from the biology of the spider’ (Deans 2004, 247).

14. My pairing of the narrative reconstructions and artistic practices at work within Erdrich’s fiction is, in part, informed by Laura M Furlan’s analysis of *The Antelope Wife* (Erdrich 1998). On the differences between revised editions of this novel, Furlan finds the artistry of Ojibwe beadwork to be thematically and structurally informative, as scattered beads are reassembled into new configurations (Furlan 2017, 165–6).

15. See Kemball (2020) for more on the disproportionate impact of COVID-19 on Native Americans, practices of statistical erasure and the structural difficulties faced by Native American health organisations during the first wave of the pandemic.

16. Here, I borrow sociologist Adele Clarke’s term ‘healthscape’, understanding Erdrich’s novel as part of a larger body of representations which depict ‘the many and varied sites where health and medicine are performed, […] and changing ideological and cultural framings of health, illness, healthcare, and medicine’ (Clarke *et al*. 2010, 105).
